# Multifactorial Prevention of Cardiovascular Disease in Patients with Hypertension: the Cardiovascular Polypill

**DOI:** 10.1007/s11906-016-0648-3

**Published:** 2016-04-15

**Authors:** M. Lafeber, W. Spiering, F. L. J. Visseren, D. E. Grobbee

**Affiliations:** Julius Center for Health Sciences and Primary Care, University Medical Center Utrecht, Utrecht, The Netherlands; Department of Vascular Medicine, University Medical Center Utrecht, Utrecht, The Netherlands

**Keywords:** Polypill, Fixed-dose combination therapy, Combination pill, Adherence, Hypertension, Cholesterol, Prevention

## Abstract

Hypertension is a major, if not the most important, contributor to the disease burden and premature death globally which is largely related to cardiovascular disease. In both the primary and the secondary preventions of cardiovascular disease, blood pressure (BP) targets are often not achieved which is similar to achievement of cholesterol goals. Combining aspirin, cholesterol and blood pressure-lowering agents into a fixed-dose combination pill called the cardiovascular polypill has been proposed as complementary care in the prevention of cardiovascular diseases in both the primary and secondary preventions of cardiovascular disease. This review article focuses on the potential role of fixed-dose combination therapy in the treatment of hypertension, outlines the pros and cons of combination therapy and emphasizes the rationale for trialling their use. Current and planned future cardiovascular polypill trials are summarized, and the prerequisites for implementation of the polypill strategy are described.

## Introduction

Hypertension is a major, if not the most important, contributor to the disease burden and premature death globally which is largely related to cardiovascular disease [1]. In the last decades, the number of individuals with uncontrolled hypertension has increased to approximately one billion [2]. Even among identified and treated individuals, a large part of them fail to reach currently recommended blood pressure (BP) targets in high-income countries [3]. The majority of these individuals have mild hypertension (grade I) without manifest vascular disease [4]. However, risk factors for cardiovascular disease such as hypertension, dyslipidaemia, obesity and insulin resistance tend to cluster, leaving these individuals prone to develop cardiovascular disease [5]. In the Western world, cardiovascular disease affects half of all individuals over their lifetimes [6]. More strikingly, the burden of cardiovascular disease is increasing disproportionally in low- and middle-income countries (LMICs), in which over 80 % of the global cardiovascular deaths occur [7, 8]. In those affected by cardiovascular disease, risk factors such as hypertension modify the risk of recurrence of a major cardiovascular event. Although rates of hypertension awareness and treatment have improved over the past few decades, control of BP in secondary prevention has similar limited success as in primary prevention. Comparable results have been seen for other risk factors such as dyslipidaemia [9•]. To call a halt to the growing incidence of cardiovascular disease will require addressing the societal determinants of the root causes of cardiovascular disease, the development of risk factors among individuals and the multifactorial use of medication to treat hypertension and other cardiovascular risk factors simultaneously [10]. A multidrug fixed-dose combination pill might be a useful tool against cardiovascular disease. This article aims to review a potential role for the polypill in the treatment of hypertension.

## Multidrug Treatment for Blood Pressure Reduction

BP-lowering therapy is one of the cornerstones of the prevention of cardiovascular disease as it reduces the risk of cardiovascular disease largely. A reduction of 10 mmHg systolic BP has been shown to lower the risk of coronary events by 20 % and of cerebrovascular events by 45 % in those without cardiovascular disease. Similarly, in secondary prevention, the risk of a recurrent coronary event is reduced by approximately 25 % and recurrent cerebrovascular event by 35 % per 10 mmHg systolic BP [11]. A single BP-lowering agent in a standard dose, in general, reduces the systolic BP by 8 to 10 mmHg. The effect is largest in those with higher pretreatment BP levels [12].

Strategies to improve BP control include the prompt shift from monotherapy to multidrug therapy [13]. In most individuals with hypertension, combining BP-lowering drugs is necessary to achieve adequate BP reductions. The rationale of combination therapy lies in an additive BP reduction when combining various classes of BP-lowering agents [12, 14]. Furthermore, multidrug therapies in a low therapeutic dose are generally better tolerated than respective monotherapies in higher dose for obtaining similar BP reductions. Accordingly, recent guidelines advocate multidrug therapy with a combination of two BP-lowering agents as an initial therapy for the majority of hypertensive patients even though this is known to be associated with diminishing adherence and inadequate prescription [15, 16]. If provided in a single pill, in addition to the potential synergistic actions, a multidrug strategy might improve patients’ adherence to medication by reducing pill burden and dosing frequency [17, 18]. European guidelines even suggest fixed-dose combinations of BP-lowering drugs over separate BP-lowering agents due to the additional benefits on adherence [15]. Therefore, fixed-dose combination (FDC) pills are well accepted in the treatment of hypertension.

## Multifactorial, Multidrug Treatment for Risk Reduction

BP-lowering therapy is one of the cornerstones of the prevention of cardiovascular disease. Additionally, anti-platelet and cholesterol-lowering therapy reduce the risk of cardiovascular events. Combining these agents into a single pill has been suggested many years ago. The concept of ‘aspolol’ (aspirin and atenolol) was first discussed in the 1970s (R. Peto, personal communication), and patents for multidrug combination pills began to be lodged in the late 1990s [19, 20]. The first major scientific meeting on this concept was held in 2001, when the World Health Organization and the Wellcome Trust initiated a meeting of experts to discuss evidence-based and affordable interventions for non-communicable diseases [21]. The term ‘polypill’ was introduced with the publication of Wald and Law’s papers in 2003. They proposed a strategy in which everyone aged 55 and older and everyone with existing cardiovascular disease would be treated with a single pill containing folic acid, aspirin, a statin and three low-dosed BP-lowering agents. By simultaneously addressing multiple cardiovascular risk factors in a low-risk population, regardless of pretreatment levels, the risk of major cardiovascular events could be reduced tremendously [22]. Instantly, large resistance arose as the lifetime use of drugs in a population at a low absolute risk of cardiovascular disease would largely promote unnecessary medicalization, adverse events and inducing a sense of protection, thereby deflecting attention from healthy behaviours. Even though combining multiple well-established cardiovascular drugs into a single polypill might result in a significant reduction of cardiovascular events in a low-risk population, this strategy is not likely to be implemented in the next decade as it would require a paradigm shift of preventive care.

Nonetheless, the concept of a multifactorial, multidrug approach regardless of pretreatment risk factor levels to reduce the risk of cardiovascular disease could be applied to populations at increased risk of cardiovascular disease, such as patients with hypertension or established cardiovascular disease. Among people without established cardiovascular disease, there has been a transition in recent decades from treatment recommendations for BP-lowering therapy and statins being based on single risk factors, e.g. BP thresholds, to treatment based on a predicted absolute risk of cardiovascular disease [23]. In these patients, multiple drugs generally are indicated and the margin of benefit is high. Regardless of initial low-density lipoprotein (LDL) cholesterol, prescribing a statin reduces the risk of a future event [24]. The reduction of LDL cholesterol is proportional to the clinical benefits [11, 25]. Similarly, for patients with mainly dyslipidaemia, most recent data from the SPRINT trial suggested that intensive BP with lower treatment goals in non-diabetic patients reduces the risk of cardiovascular disease even further [26•]. The use of aspirin in primary prevention is still under debate and is generally not recommended, although recent evidence, demonstrating a potential reduction in cancer deaths with long-term use, might change the risk/benefit equation [27, 28].

Regardless of the exact components of polypills, an FDC pill could be considered as a multifactorial baseline therapy providing the minimum standard therapy for moderate- to high-risk individuals with additional benefits on adherence. The principal goal of a polypill strategy would be reducing the risk of major cardiovascular events and mortality, and not necessarily normalizing risk factors or reaching treatment goals. However, this strategy does not rule out tailored care as every individual can be treated with additional BP- and/or cholesterol-lowering agents if the treatment goals are not achieved.

Similar to FDC pills with solely BP-lowering agents, improvement of adherence in patients at risk of cardiovascular disease is an important principle of the polypill concept. Long-term adherence is low, with only 45 % adherence to BP-lowering therapy and statin use after 12 months [29]. Combination pills have been shown to increase this adherence by reducing the number of pills and providing simplicity in the treatment [30].

Another major advantage of a polypill with major consequences on accessibility to healthcare in developing countries relates to the low costs and improved affordability. By dispensing a single generic pill with multiple BP-lowering agents and a statin for hypertension instead of the individual drugs, packaging, dispensing and pharmacy expenditure can be reduced enormously. Hence, the concept of the polypill was proposed as a simple, innovative and cost-effective public health strategy to influence accessibility to medications and adherence to treatment at a global scale.

There are also certain drawbacks to an FDC pill, meaning that a polypill strategy cannot be applied in every individual. Due to fixed combinations in a single pill, there is no flexibility in being able to change the class of BP-lowering drugs due to contraindications or unacceptable adverse effects. In particular, patients with primarily hypertension might cease using an FDC pill with BP-lowering agents due to statin-associated myalgia. These issues might be addressed in the future by the marketing of several FDC pills with various components, thereby giving the clinician greater choice of drug class whilst retaining the convenience of a polypill. In addition, FDC pills with two, three or four BP-lowering agents and a statin might be formulated for hypertensive patients in order to limit the number of pills whilst achieving BP goals.

## Clinical Evidence of FDC Pills

FDC formulations correspond closely to combinations that are already in widespread use, such as an ACE inhibitor, thiazide diuretic, beta-blocker and statin. The generic drugs used as components of an FDC pill have been marketed for many years in the prevention of cardiovascular disease in both primary and secondary preventions. It may very well be that many patients use the identical components as a polypill administered at the same time, although not in a single pill or capsule. However, substantially different from when using an FDC, each of the individual components in standard clinical practice is generally prescribed at the discretion of the treating physician for a specific indication and taking into account relative contraindications. The concept of the polypill includes promoting a widespread use of multifactorial cardiovascular risk-lowering treatments regardless of risk factor levels which need to demonstrate beneficial effects in trials.

One of the first clinical trials was a randomized, double-blind, placebo-controlled trial in Iran. A total of 475 low-risk participants without cardiovascular disease or cardiovascular risk factors, aged 50 to 79 years, were randomized to an FDC pill (aspirin 81 mg, enalapril 2.5 mg, hydrochlorothiazide 12.5 mg and atorvastatin 20 mg) or placebo for a period of 12 months. The trial showed that the FDC achieved modest reductions of BP when treating a baseline systolic BP of 125 mmHg (mean difference systolic 4.5 mmHg and diastolic 1.6 mmHg) and LDL cholesterol (baseline LDL cholesterol 3.0 mmol/L, mean difference 0.46 mmol/L) even though the FDC pill was well tolerated [31].

In addition, in the randomized, double-blind, placebo-controlled ‘Programme to Improve Life and Longevity’ (PILLpilot) trial, 378 individuals at intermediate risk of cardiovascular disease were randomized to using an FDC (aspirin 75 mg, lisinopril 10 mg, hydrochlorothiazide 12.5 mg and simvastatin 20 mg) or placebo during 12 weeks. The baseline systolic BP was 134 mmHg and the LDL cholesterol was 3.7 mmol/L. Using the polypill resulted in a 10 mmHg (95 % CI 8 to 12) lower mean systolic BP and 0.9 mmol/L (95 % CI 0.8 to 1.0) lower mean LDL cholesterol compared to using placebo [32]. The effect of the polypill on risk factor levels was modified by the baseline levels of these risk factors, resulting in the largest BP reduction in those with hypertension. The achieved cardiovascular relative risk reduction was only modestly modified by the baseline levels of these risk factors. Although mild adverse events such as cough and hypotension were reported more often in the polypill group, these were not related to baseline risk factor levels, suggesting that patients with mildly increased risk factor levels, but an overall raised cardiovascular risk, would also benefit from being treated with a polypill [33].

Recently, a dosage study has been performed in which the polycap (aspirin 100 mg, atenolol 50 mg, hydrochlorothiazide 12.5 mg, ramipril 5 mg and simvastatin 20 mg) was used in patients with a baseline systolic BP of 144 mmHg and LDL cholesterol of 2.4 mmol/L. ‘The Second Indian Polycap Study’ (TIPS-2) with 518 patients demonstrated that a double-dosed polycap with potassium supplementation resulted in a systolic BP reduction of 17.4 mmHg which was a 2.8 mmHg additional systolic BP reduction compared to a single-dosed polycap. The LDL cholesterol was only 0.3 mmol/L decreased by using a double-dosed polycap versus 0.1 mmol/L when using a single polypill. It should be noted that at baseline, most patients already used a statin. Both treatments had similar discontinuation rates (7.8 % in the double-dosed group versus 6.9 % in the single-dosed group), suggesting also a potential role for various dosed FDC pills [34].

As one of the most fundamental evidence are comparative clinical studies of a polypill-based treatment strategy versus reference treatment, these are necessary in order to put into perspective the improvement obtained with a polypill-based treatment strategy compared to usual care. Only confirmatory trials are able to show the net effect of the various hypothesized benefits of a polypill in a real-life population. The acceptability, efficacy and economic impact of a polypill-based strategy for the prevention of cardiovascular events are likely to vary substantially between countries, and these will be greatly influenced by the existing health-care systems i.e. usual care and the subsidies offered for drug therapy. Hence, information about these health-care system parameters for implementing a polypill-based strategy from both developed and developing countries in high-risk patients is imperative. It could be hypothesized that implementation of the polypill strategy would be most beneficial in LMICs.

A large international initiative to address the effects of polypill versus usual care was undertaken by the ‘Single Pill to Avert Cardiovascular Events’ (SPACE) Collaboration. The Collaboration was initiated in 2009 and comprises a group of academic investigators from Australia, New Zealand, India, China, South Africa, Brazil, Canada, the UK, Ireland and the Netherlands. Currently, three trials with similar design have been published [30, 35, 36]. Each trial is as similar to *real life* as possible within each national setting, whilst maintaining as much uniformity between all trials as possible to facilitate the final pooling of data. The ‘Use of a Multidrug Pill In Reducing cardiovascular Events’ (UMPIRE) trial was the first randomized, clinical trial comparing a polypill-based treatment strategy for the delivery of medication (aspirin, two BP-lowering agents and a statin) to usual care among participants with established cardiovascular disease or at equivalent high risk (an estimated 5-year cardiovascular risk of ≥15 %, e.g. hypertensive patients) in India and three European countries (the UK, Ireland and the Netherlands). In the FDC group, physicians could use a polypill that contained aspirin 75 mg, simvastatin 40 mg, lisinopril 10 mg and either atenolol 50 mg or hydrochlorothiazide 12.5 mg. In the usual care group, treatment continued according to the physicians’ discretion. In total, 2004 participants were randomized in India and Europe. After a median follow-up of 15 months, the polypill group showed to have an improved adherence (relative risk of being adherent 1.33; 95 % CI 1.26 to 1.41) with a concurrent 2.6 mmHg (95 % CI 1.1 to 4.0) lower mean clinic systolic BP and 0.11 mmol/L (95 % CI 0.05 to 0.17) lower mean LDL cholesterol compared to the usual care group [30]. The size of these benefits was regarded as modest in the relatively well-treated high-risk population. As participants were randomized to continuing usual care or a polypill, the consequences of switching to a polypill were likely to be influenced by medications and doses used at baseline [30]. In the ‘IMProving Adherence using Combination Therapy’ (IMPACT) trial, 513 patients in New Zealand were randomized to an FDC-based care or usual care, similar to the UMPIRE trial. In this trial, there was no statistically significant improvement in systolic BP (mean difference −2.2 mm Hg; 95 % CI −5.6 to 1.2) or in LDL cholesterol (mean difference −0.05 mmol/L; 95 % CI −0.17 to 0.08) after 12 months of treatment with the polypill or usual care [35]. In the ‘Kanyini-Guidelines Adherence with the Polypill’ (GAP) trial, 623 Australian patients were included. After a median of 18 months, the polypill-based strategy did not show a differences in systolic BP (mean difference −1.5 mmHg; 95 % CI −4.0 to 1.0) or total cholesterol (mean difference 0.08 mmol/L; 95 % CI −0.06 to 0.22) [36].

Data of these three trials have been combined into a prospective, individual participant data meta-analysis. Compared to usual care, participants with the FDC pill had higher adherence to combination treatment (relative risk 1.58; 95 % CI 1.32 to 1.90), a 2.5 mmHg (95 % CI 0.4 to 4.5) lower systolic BP and 0.1 mmol/L (95 % CI 0.0 to 0.2) lower LDL cholesterol. Furthermore, baseline treatment levels were a major effect modifier for adherence and systolic BP with greatest improvements seen among those under-treated at baseline [37•].

## Marketing the Polypill

Licensing is an essential step for marketing combination therapy in any population. Regulatory agencies are faced with new issues when evaluating novel FDC formulations. The US Food and Drug Administration (FDA) and the European Medicines Agency (EMA) have approved various two-, and a couple of three-drug combinations, but neither have granted a marketing license for four- or five-drug formulations. Additionally, previous combination treatments only addressed one risk factor, i.e. hypertension, complicating decision-making as in the polypill concept, multiple risk factors are addressed simultaneously, irrespective of risk factor levels. Currently, effects of FDC formulations have been shown in various comparative trials powered on risk factors. Although the emerging opinion suggests that cardiovascular endpoint trial studies are not required, this is not yet undoubtedly adopted by the regulators. Treatment effects on established surrogate endpoints that are reasonably likely to predict clinical benefit, such as LDL cholesterol and systolic BP, may result in marketing authorization [38].

## Focus on High-Risk Patients

Given the present data on the effect of a polypill in moderate- to high-risk patients, it is highly likely that a polypill-based treatment strategy will be adopted in the forthcoming years. Especially in LMICs, a polypill is, in general, the best alternative treatment compared to hardly any treatment. Yet, similar in the Western countries, the polypill has shown to have beneficial effects on adherence and cardiovascular risk factor levels, indicating a role for the polypill as a complementary treatment strategy in the prevention of cardiovascular disease. However, if FDC formulations are licensed and a polypill-based treatment strategy is to be successfully implemented, health-care professionals will need to be convinced of the benefits of this approach. In general, patients largely prefer using a polypill over usual care [39]. Currently, only one polypill has been marketed [40•].

## Reservations for a Low-Risk Population

There is a theoretical rationale for a polypill-based treatment in a low-risk population, in which imperfect and expensive screening is avoided. Whilst the low-risk population has a small absolute risk of cardiovascular events, this population includes most of those who will experience cardiovascular events due to the great size of a low-risk group [22]. Additionally, with the present increasing incidence of cardiovascular disease, there would be insufficient physicians and health-care workers worldwide to screen and treat every individual at risk. Instead of aiming lifestyle first and pharmaceutical treatment only if required, a multifactorial approach to prevent and treat cardiovascular diseases is far more efficient. Although lifestyle modification is natural and safe, it is generally not low-cost, not simple and not sustainable [41]. Yet, even a decade since the introduction of the concept, there appears to be little support for a polypill-based preventive strategy in a low-risk population. Possibly, if trials involving moderate- and high-risk individuals show clear beneficial effects, the idea of offering treatment to everybody older than 50 years might raise widespread interest.

Some trials have been initiated in a low-risk population with regard to risk factor reductions [31, 42]. Nevertheless, licensing a polypill for a low-risk population will undoubtedly require large clinical endpoint trials. The ‘Heart Outcomes Prevention and Evaluation 4’ (HOPE-4) study aims to evaluate the effect of a polypill based-treatment strategy (simvastatin 20 mg, ramipril 5 mg, atenolol 50 mg and hydrochlorothiazide 12.5 mg) on major cardiovascular endpoints compared to usual care in approximately 9500 participants aged 50 years and older. Similarly, ‘The International Polycap Study 3’ (TIPS-3) aims to evaluate the effect of the polycap in double strength (simvastatin 40 mg, ramipril 10 mg, atenolol 100 mg and hydrochlorothiazide 25 mg), aspirin and cholecalciferol on major cardiovascular events in 5500 participants at intermediate risk aged 55 years and older in a factorial design. The results of both trials are anticipated to become available by 2020.

As an alternative option for a licensed polypill-based treatment strategy, alternatives have been marketed. In the early 2012, Wald and Law performed a randomized crossover trial including 86 individuals aged older than 50 years. At baseline, the systolic BP was 143 mmHg and the LDL cholesterol was 3.7 mmol/L. The participant received the polypill (simvastatin 40 mg, losartan 25 mg, amlodipine 2.5 mg and hydrochlorothiazide 12.5 mg) or placebo during a period of 12 weeks and switched to the alternative treatment for another 12 weeks. The use of the polypill resulted in a 17.9 mmHg (95 % CI 15.7 to 20.1) lower mean systolic BP and 1.4 mmol/L (95 % CI 1.2 to 1.6) lower mean LDL cholesterol. They suggested that long-term reduction of this magnitude would have a substantial effect on preventing cardiovascular disease [43].

## Conclusion

Currently, BP-lowering therapy in hypertensive patients aims to reduce the risk of cardiovascular disease. The polypill could be an important tool to reduce the risk of cardiovascular disease by simultaneously treat multiple risk factors and not exclusively hypertension. Data indicate that combination pills can produce sizeable risk factor reductions and increase long-term adherence to therapy. Overall, a polypill is preferred by patients over separate pills and the therapy is low-cost. Results from ongoing trials that are further assessing the effectiveness of combination pills in reducing BP levels and cholesterol and the effects on adherence to indicated medications and clinical outcomes would provide clear evidence on the role of polypill-based treatment strategy on the long run. It would also hold implications for policy-making to address both primary and secondary cardiovascular preventions globally.
